# Experiences of Birth during COVID-19 Pandemic in Italy and Spain: A Thematic Analysis

**DOI:** 10.3390/ijerph19127488

**Published:** 2022-06-18

**Authors:** Sofia Colaceci, Gloria Anderson, Veronica Ricciuto, Denise Montinaro, Giorgia Alazraki, Desirée Mena-Tudela

**Affiliations:** 1 Department Faculty of Medicine and Surgery, Saint Camillus International University (UniCamillus), Via di Sant’Alessandro 8, 00131 Rome, Italy; veronica.ricciuto@unicamillus.org; 2 Department of Biomedicine and Prevention, University of Rome Tor Vergata, Via Montpellier, 1, 00133 Rome, Italy; andersongloria0@gmail.com; 3 Associazione Rinascere al Naturale, Via Umberto I, n. 42, 73021 Calimera, Italy; mdenise555@yahoo.it; 4 Independent Researcher, 00133 Rome, Italy; galazraki@yahoo.it; 5 Department of Nursing, Universitat Jaume I, Avenida de Vicent Sos Baynat, s/n, 12071 Castellón de la Plana, Spain; dmena@uji.es

**Keywords:** childbirth, women, midwifery, obstetric violence, COVID-19, qualitative research

## Abstract

Becoming parents during the pandemic of coronavirus disease 2019 (COVID-19) has been a challenge. The purpose of this study was to describe the impact of the pandemic on new and expectant parents in both Italy and Spain. A descriptive qualitative study was carried out by collecting social media posts written by parents between March 2020 and April 2021. The posts were inserted in a data collection form and assessed separately by two authors. The coding was performed manually using the long table analysis method and a thematic analysis was performed. Three main themes were identified: (1) care; (2) overcoming difficulties and problem-solving strategies; and (3) legislation and anti-COVID-19 measures. The main issues for parents were the limited access of partners to antenatal care services and mother–newborn separation. Due to restrictive measures, many parents adopted different coping skills. Some hospitals were able to maintain high standards of care; however, a lot of discretion in legislation and the application of anti-COVID-19 measures in healthcare services was perceived by parents. The COVID-19 pandemic has heavily affected the way parents experienced pregnancy and birth. Becoming parents during the pandemic has exacerbated some fears that usually characterize this event, but it has also triggered new ones, especially in the first months.

## 1. Introduction

The COVID-19 pandemic has been a challenge for public health worldwide. It is estimated that, from the first case in 2020 to the 30th of June 2021, COVID-19 has affected about 48 million people in Europe, of which over 1 million have died [1]. Italy and Spain are among the top six European countries for the number of infections and deaths [2].

The pandemic has significantly impacted different aspects of everyday life, especially during lockdowns, and also forced a reorganisation of all health services, including those of the birth process in Europe [3]. In fact, although the pandemic has inevitably given society pause for thoughts on the concept of memento mori, life did not stop and, therefore, women have continued to give birth and children to be born. In the first emergency phase, the reorganisation of health services was based on the availability and logistics of resources and on risk plausibility because the scientific evidence [4,5] was still limited and sometimes inconsistent. The organisational models and clinical practices of the birth process have been progressively adapted to the increasing evidence and also thanks to the synergistic collaboration among institutions and the scientific community [6,7]. In particular, in Spain, the Ministry of Health published a technical document on the management of pregnant women and infants born during COVID-19 [8]. In Italy, the National Institute of Health coordinated the publication of an evidence summary and related indications through a periodically updated report addressed to health professionals and health decision-makers to support them in the maternal–child care and organisational choices [5,9].

Pregnancy is a life stage which implies profound changes in a woman’s identity and self-perception [10]. Worries about bodily changes and physical symptoms, fear of childbirth, and childcare responsibilities can affect pregnant women’s well-being [11]. Becoming parents during the pandemic has exacerbated these fears and also triggered new ones, especially in the first lockdown months, when information was still scarce [12]. The measures adopted by hospitals to apply national and international guidelines has presumably impacted new and expectant parents’ experience during the pandemic. Although Inversetti et al. found no differences in the satisfaction level of the birth event between women who gave birth before and those who gave birth during the pandemic [13], Bartick et al. found that 27.9% of mother–infant dyads with confirmed or suspected COVID-19 infection were separated soon after birth and 58% of mothers experienced this with anxiety and stress [14]. A qualitative study was therefore conducted with the aim of describing parents’ experiences regarding pregnancy and birth during the COVID-19 pandemic in Italy and Spain.

## 2. Materials and Methods

### 2.1. Design

We conducted a descriptive qualitative study [15] performing a thematic analysis to answer the research question: what were parents’ experiences regarding pregnancy and birth during the COVID-19 pandemic in Italy and Spain?

### 2.2. Data Collection

The study was carried out between April and June 2021 by retrieving shared experiences through social media posts between March 2020 and April 2021 by Italian and Spanish new or expectant parents. Italy and Spain were chosen because they were involved in the epicentre of the first COVID-19 wave. More specifically, we referred to the leading Italian and Spanish associations dedicated to the rights of women and newborns (Rinascere al Naturale and El Parto es Nuestro). In order to provide useful information to expectant parents, the associations invited parents to report their experiences, especially the critical issues experienced during pregnancy and childbirth, by sending a written private message. The associations then published the anonymous posts on the public section of their websites and Facebook pages.

Researchers extracted these public posts from these Facebook pages, informing the association boards, which did not raise objections in this regard given the public domain of anonymous posts.

We considered all the posts published between March 2020 and April 2021. Posts were collected regardless of parity because both those referring to the birth of the first child and subsequent children were assessed as equally valid. The written posts were inserted in a data collection form in a text file and structured in different identification fields: publication date, extraction link, full text, number of reactions, and number of comments. Two authors proceeded to separately assess the collected posts, selecting those pertinent to the study objective. More specifically, we included posts concerning experiences (approach of healthcare services, emotions, fears, and doubts) lived by parents during pregnancy, birth, or post-partum by new or expectant parents during the COVID-19 pandemic. We excluded posts not related to the pandemic (e.g., experiences dated back to past years), lacking in experiential content (e.g., interventions encouraging women or parents or posting scientific updates), and not written by parents (e.g., healthcare professionals).

### 2.3. Data Analysis

The posts selected were subjected to thematic analysis to understand experiences, thoughts, or behaviors through a data set to identify patterns in meaning, according to Braun and Clarck [16]. The coding was performed manually through the long table analysis method [17]. The emerged themes were tested to verify their validity by two authors, referring to an external researcher in case of disagreement. We used the SRQR checklist to describe the study [18]. To keep maximum adherence to the original posts, we have inserted some verbatim translated quotes.

### 2.4. Ethics

According to the Italian (GU n. 76 of 2008) and Spanish regulations (Organic Law n. 3 of 2018) about non-interventional observational studies, approval by the Ethics Committee was not requested. Since the two social media sites publish anonymous posts, the authors did not request the consent of the parents. Furthermore, the latter were aware that their posts would be posted on a public page anonymously, in accordance with the information on the related Facebook pages. However, the authors were careful to select texts not containing personal information. The symbol “(…)” was used to omit unnecessary information or information reporting personal data (e.g., baby or hospital name).

## 3. Results

The total number of selected posts is 236: 196 Italian posts and 40 Spanish posts. For the purpose of the thematic analysis, applying the criteria listed above, we selected 128 Italian (IT) and 34 Spanish (SP) posts. The posts included were published between July 2020 and April 2021.

**Figure 1:**
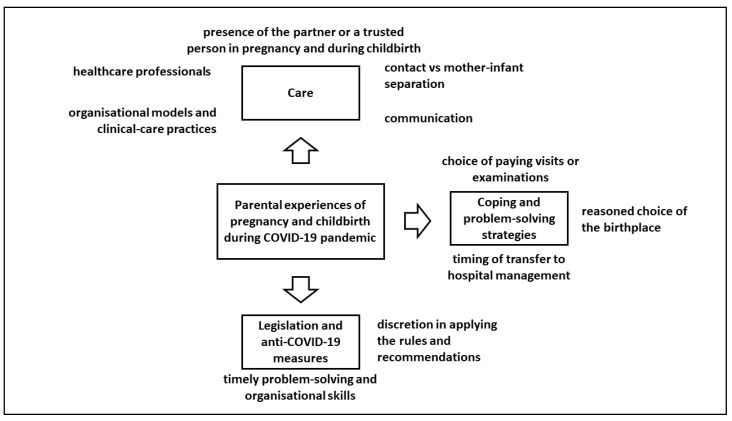
Main findings.

Three main themes were identified: (1) care, (2) overcoming difficulties and problem-solving strategies, and (3) legislation and anti-COVID-19 measures (Figure 1).

### 3.1. Theme 1 Care

With regard to care, five sub-themes emerged: organisational models and clinical-care practices, healthcare professionals, presence of the partner or trusted person during pregnancy and during childbirth, continued contact vs. mother–infant separation, and communication.

#### 3.1.1. Organisational Models and Clinical-Care Practices

The hospital organisational models applied during the pandemic also impacted birth experiences in relation to the logistical/structural availability of the hospitals. In fact, where it was possible, there was a separation between COVID-19-positive and -negative processes, where healthy women were able to have greater freedom of movement and fewer restrictions.

*“The (birth) unit was divided in 3 zones (…), one in each floor. Once I arrived (…) they took me to the orange area to do and wait for the swab result; it came out negative, so they took me to the white zone. If the swab would have been positive they would have taken me to the red zone, together with other positive women, and also there I could have kept my daughter with me.”*

(7/03/2021, IT)

*“Our birth unit has become mixed… half of it was ‘normal’ maternity unit and the other half was ‘COVID’ maternity unit… so us, healthy women, we were in our room and we couldn’t even go out to the corridors.”*

(3/03/2021, IT)

Clinical practices heterogeneously respected women’s needs and international recommendations, ranging from more or less restrictive levels in terms of adopting anti-COVID-19 measures.

*“The delayed cord cutting/clamping was practiced without even asking for it, breastfeeding was immediately encouraged, and skin-to-skin was proposed. They did not take the baby away to wash or dress (…) The staff is extremely attentive to physiology and to the mother and child needs.”*

(4/02/2021, IT)

*“The protocol of my hospital (…) says that COVID-19 positive mothers will give birth alone (…) and the baby will be separated at birth. The mother will go to the University Hospital and the baby will be brought to the Neonatal Hospital.*

(777, 2020, SP)

*In the hospital there were no rooms for women who gave birth, so they had to stay in the birth unit, occupying the delivery rooms (…), where there is a swimming pool and where I could have delivered. So, this affected my birth, because I couldn’t use it.”*

(797, 2020, SP)

In some posts, both Italian and Spanish women reported that their dissatisfaction with the childbirth experience was not due to the COVID-19 measures but to the usual care which was not in line with international recommendations even before the pandemic.

*“I’m totally sure that not all of what happened during labour and birth, and what I’m complaining about, has to do with COVID-19. I think it is mostly how they usually proceed and I think they should change it.”*

(825, 2020, SP)

#### 3.1.2. Healthcare Professionals

Being under the care of empathetic health professionals represented a recurring theme. The importance of the health personnel’s support emerged, especially when having a partner or a trusted person close to the woman was denied.

*“Midwives’ care was super dedicated, kind, sweet, affectionate, attentive … They helped me a lot with breastfeeding, and I think that part of my wellbeing about the birth is thanks to them.”*

(801, 2020, SP)

Women subjected to C-section more frequently reported discontent, as sometimes the staff did not support them in the post-partum period with advice about hygiene or breastfeeding.

*“After the caesarean I was exhausted (…) They came to leave the food on the table and go away, despite the calls for help (…), nobody ever come. They brought me the baby after 12 hours from birth and it was very difficult to breastfeed or change position without any support (…). If you do the caesarean in COVID time, I think you are penalised double.”*

(27/07/2020, IT)

#### 3.1.3. Presence of the Partner or a Trusted Person

One of the most recurring themes is the one concerning the presence of the partner or a trusted person during checks in pregnancy, but especially during labour and birth. Several positive posts were written on hospitals allowing access during labour, birth, and rarely, during the hospitalisation.

*“First of all, the presence of the father is always guaranteed. He was with me for the whole labour. He was in the delivery room, then for two hours after birth, just the three of us, with the baby resting on my belly; and then he could come every day visiting from 7 to 20, without limitations.”*

(4/02/2021, IT)

Many other posts reported the application of restrictions on the partner’s presence. In some hospitals, especially during the first wave, the ban given to COVID-19-positive women of having a person by their side was extended to all women.

*“I had to say goodbye to my husband on the stairway of the hospital, where he left me with my trolley. I remember a lot of fear and sorrow because I imagined our first birth different, experiencing the delivery together from labour until the encounter with our baby.”*

(2/08/2020, IT)

*“Am I going to be alone in this horrible situation? Who will calm me down and support me? Him alone?? Is he going to be alone, without knowing how I feel? Without being able to meet his child since the first second of life? (…) They took away consultations, birth course, we are already quite alone, do not take away the support of our partners, the best persons able to reassure and support us …“*

(771, 2020, SP)

In other hospitals, access was allowed after having ascertained the infectious state of the woman and her partner, who were considered as suspected cases until the swab result arrived.

*“They did not let the father in… I had to insist to let him in to see the baby for few minutes … The nurse said she let him in secretly (…) The problem was that the result of my swab was not ready.”*

(20/07/2020, IT)

Some couples had the opportunity to stay in contact via video call during the birth.

*“I had the luck to talk to my husband and have a video call with him during the birth.*

(6/02/2021, IT)

*In the birth plan I asked that the father be able to watch the expulsive by video call. I insisted on it and they said not to worry. There was no video call, the circumstances did not allow it… It pained me!”*

(786, 2020, SP)

The restrictions on visiting hours in the maternity unit and access to the delivery room caused discomfort to the partners, especially when the hospital was very far from home.

*“I went with my wife to the hospital for a check and obviously I waited outside in the parking lot … she called me and said they would have hospitalised her straight away… (…) Then, the ordeal started … every day 60 km for less than one-hour visit.”*

(22/09/2020, IT)

In other cases, the greatest inconvenience was due to the lack of waiting rooms for fathers, who were often forced to wait in the car or in the corridor, according to security discretion. Often the father was considered a mere visitor without rights in the birth process, both during pregnancy and childbirth (access to prenatal visits was prohibited, and during labour and childbirth the father’s presence was limited or denied).

*“I was waiting in the little corridor between the ward and the delivery room. In absolute silence no one (…) updated me on anything. Suddenly a scream. Shivers. And finally, the cry of a newborn. My son! Interminable minutes passed before I could see my son coming out… and a doctor told me: “You have 5 minutes to take pictures and fill up the modules”. (…) I asked about my wife but nothing. I waited, but again they threw me out of the ward. I finally saw her passing by the corridor, having the time to say: “Everything ok”, then she disappeared behind the door of the ward.”*

(22/09/2020, IT)

#### 3.1.4. Contact vs. Mother–Infant Separation

Another common aspect observed was the mother–child separation at birth. Despite the positive experiences concerning hospitals which have always guaranteed the contact of the dyad, many posts reported women complaining of a long separation: they were able to have the child only after many hours of childbirth, sometimes due to waiting for COVID swab results.

*“I gave birth at 1 am and I saw the baby in the nurse arms from a distance. They took him away and brought him back to me only at 11 am … That night, although I was very tired, I couldn’t sleep and I kept walking up and down in the room … It was endless!”*

(3/03/2021, IT)

*“A doctor (…) told us that as a consequence of COVID-19 measures, there was not a place dedicated to skin-to-skin contact with babies. In other places they allow the mother—even after a caesarean section—to do skin to skin and, however, in this hospital that possibility is not contemplated, neither by taking it away from the parents due to COVID-19.”*

(825, 2020, SP)

In other cases, the separation was due to the post-partum care of women who experienced a C-section; since it is difficult to manage a newborn for them, practical organisations did not guarantee rooming-in service.

*“I couldn’t keep the baby with me because I had the catheter, and since the father was not allowed because of COVID, they couldn’t leave my baby with me. I was not ready for this, and when they took her away I felt dying inside and this is how I still feel today for not having the possibility to hold her when she needed it most.”*

(27/03/2021, IT)

Similarly, this happened when newborns needed medical care and therefore had to be kept in the neonatology unit.

*“The baby had been placed under the lamp because he had jaundice. So, I waited until late evening, slipped out of the room, left the ward, took the stairs (despite the pain due to the stitches) to avoid the elevator which COVID mothers were used to take. When I arrived at the nursery, I was stopped, reprimanded and sent back to my room … That day they didn’t let me see or breastfeed him!”*

(4/03/2021, IT)

#### 3.1.5. Communication

In several posts, communication problems emerged leading to discomfort and stress for the new and expectant parents.

*“On the third day of hospitalisation, the nurses brought all babies to their mums, except mine… Nobody told me anything, a real chaos in that hospital! Only few hours later, after a lot of stress and worries they told me he was placed under the lamp because of jaundice.”*

(4/03/2021, IT)

*“Yesterday my partner and I went to the (…) hospital because I haven’t been noticing any fetal movement in the last two days and we were worried (…). During the 1 hour and half that I was inside (…) nobody went out to inform my partner (…), not even to say that (…) everything was fine. I started crying because I asked to record the sound of the baby heartbeat to send a message to my partner and they denied it “according to COVID measures.”*

(833, 2021, SP)

### 3.2. Theme 2 Coping and Problem-Solving Strategies

Couples adopted various strategies to face the situation, including the choice of paying for private visits or exams, the reasoned choice of the birthplace, and time management regarding when to transfer to the hospital.

#### 3.2.1. Choice of Paying for Visits or Examinations

Several Italian posts reported the choice of paid visits or exams during pregnancy or after childbirth provided by private medical professionals because of the impossibility of both booking them in public health services and having the partner’s access guaranteed.

*“I am at the beginning of my pregnancy and at my first visits/ultrasounds, the first is private in order to avoid the exclusion of my partner.*

(19/08/2020, IT)

*(After childbirth, Ed.) I have never been able to do a gynaecological visit in a public hospital of the area where I live. And recently also in the far hospital where I used to go they told me they interrupted the visits due to COVID, and they guarantee the service only during pregnancy. Even if I couldn’t afford it, I am forced to do it privately, or paying the ticket (the fixed price for a medical service in the Italian public health system, Ed.).”*

(25/03/2021, IT)

#### 3.2.2. Reasoned Choice of the Birthplace

Many women made the choice of where to give birth in a different way during COVID-19, considering the type of experience they would have liked to have and the level of restrictions applied by the hospital. Therefore, some women changed hospitals during the pregnancy, opting for structures outside their province or region.

*“I should have given birth in the province of (…), but if they won’t let my husband in, I might consider to give birth in the hospital of another province that is as far as the other one.”*

(19/07/2020, IT)

Other women opted for private clinics.

*“I have been (in the clinic, Ed.) only two and a half days because I signed for the discharge, and I have also been also able to let my first child meet his little brother. I have been lucky.”*

(5/04/2021, IT)

Finally, several posts concerned homebirth. Some women chose an out-of-hospital birth regardless of COVID-19, reconfirming their choice in light of the pandemic events. Other women considered this option, but they did not opt for it due to health problems, while others raised their concerns about this option due to eventual complications.

*“I gave birth at home on the 26th of March, in the middle of the pandemic. I had already decided to give birth at home, but let’s say it was good timing. It was a beautiful experience.”*

(1/08/2020, IT)

*“I am a future mother… The idea of having to go through labour and childbirth alone destroys me… I am not enjoying the pregnancy because this is my only thought… I considered to give birth at home, especially not to be alone… But I’m scared of complications… I’m desperate, I cry night and day.”*

(1/08/2020, IT)

*“I considered a home birth, mainly because they were separating babies from mothers and leaving women without their partner due to the pandemic. Luckily, I gave birth in June and by then they stopped doing these atrocities in many hospitals.”*

(817, 2020, SP)

#### 3.2.3. Timing of Transfers to Hospital Management

From several Italian posts, it emerged that some women chose to postpone the timing of the transfer to the hospital and to spend most of the time during labour at home in order to stay as much as possible with their partners. Sometimes this choice involved arriving at the hospital at the imminence of the birth.

*“I decide to stay as long as possible at home because I was too afraid to go through labour alone (…) without the support of my husband… After about an hour of severe pain I already begun to feel the irrepressible desire to push… The car ride to get to the hospital was about an hour long (…) I arrived at the hospital fully dilated… With the baby’s head between my legs.”*

(6/02/2021, IT)

In addition, unplanned homebirths occurred because although they had gone to the hospital, they were instructed to go home and return when the contractions were stronger and regular. However, the rapid evolution of labour did not allow some of them to return to the hospital.

*“I gave birth at home, but not because I decided to. In the morning I went to the hospital and they sent me home because dilated only 2 cm, the contractions were still irregular… and the COVID situation there in the hospital… I was unable to return to the hospital and I gave birth alone without any assistance.”*

(8/02/2021, IT)

### 3.3. Theme 3 Legislation and Anti-COVID-19 Measures

Regarding the parents’ opinions of the legislation and anti-COVID-19 measures, most of the posts refer to the perception of the restriction level applied by the hospitals. Moreover, requests to the hospitals for better organisational capacity emerged with the aim of meeting the parents’ needs.

#### 3.3.1. Discretion in Applying Rules and Recommendations

Several posts focused on the restriction level of the regulations and recommendations applied by the hospitals.

*“The Local Health Service says I have to ask directly the hospital because it is at its discretion. This self-management really seems to me a way not to take responsibility or, simply, every hospital and health service manage things differently.”*

(2/09/2020, IT)

In other posts, parents highlighted some contradictions, especially those referring to the separation of mother and child or the absence of the partner compared to other gatherings occurring at the hospital.

*“There may be two couples in one room, an undefined number of medicine students, midwives and doctors inside the delivery room, but there is no place to do skin to skin, something so important for a newborn.”*

(825, 2020, SP)

#### 3.3.2. Timely Problem-Solving and Organisational Skills

Some posts expressed the need to improve the problem-solving and organisational skills of the healthcare managers to ensure parents have a better experience and more satisfaction, for example, by offering and ensuring rapid results for the COVID swabs.

*“These regulations are unacceptable! (…) Hospitals should be provided with devices for a rapid swab result! Why should I risk not to share my greatest joy with my son’s father?! ABSURD POLICY!”*

(6/07/2020, IT)

## 4. Discussion

In this study, we investigated the experiences and challenges experienced by Italian and Spanish new or expectant parents during the COVID-19 pandemic. Most of their doubts, fears, and sorrow were indirectly due to the changes to maternity care that were implemented in response to the pandemic. More specifically, the findings of this study focus on two main issues: the presence of the partner or a trusted person during labour and childbirth and mother–child contact. McKinlay et al. (2022) stated that the exclusion of the partner from the birthplace was one of the most recurrent issues reported by women during the pandemic [19]. In line with the literature [20,21], our study confirms that the partner was often considered merely a visitor instead of an active protagonist in the birth event due to the procedures adopted in the healthcare facilities. 

Concerning mother–infant contact after childbirth, it was often allowed only after a negative swab result, even though in May 2020 it was established that being a COVID-19-positive woman is not a valid reason for mother–child separation [5]. Bartrick et al. [14] showed that dyad separation can impact breastfeeding success and maternal emotional health, which was already at risk during the pandemic. Similarly, Molgora and Accordini showed a negative impact on women’s well-being during pregnancy and after childbirth caused by the pandemic and regulations to contain it, representing an additional risk factor for this specific population [22].

Women’s dissatisfaction with maternity services during the pandemic was mostly associated with COVID-19 restrictions which resulted in prenatal health checks and birthing classes being cancelled, postponed, or performed through telemedicine clinics [23]. It has already been reported in the literature on childbirth satisfaction [24,25] and obstetric violence [26,27] before the COVID-19 pandemic that birth experience is influenced by factors such as lack of support from health professionals, perception of disrespect for expressed needs, and ineffective communication. Under ordinary conditions, these factors are known to be a disturbing interference which could impact the expected evolution of the physiological process of birth and the post-partum period, including lactation. Starting to breastfeed and maintaining its effectiveness can be challenging because of socio-cultural factors [28], the education or attitudes of healthcare personnel [29], and formula prescription during hospitalisation, mostly without evidence-based clinical reasons [30]. During the pandemic, difficulties with latching were associated with a lack of professional support, especially in the case of first children or women subjected to C-sections [31,32]. On the contrary, distance working and the opportunity to be with the infant all the time during lockdown were mentioned as some facilitators that had a positive impact on breastfeeding [33].

The literature reports that parents’ coping strategies adopted during the pandemic to manage stress were in line with those previously reported before the pandemic [23]. However, parents-to-be carefully evaluated the options for the birthplace and birth preferences [34]. In this regard, the phenomenon of “obstetric tourism” or “birth tourism”, outside of the province or outside of the region, is an exciting finding that emerged from our study. Similarly, some couples opted for or would have opted for a private clinic or a homebirth for two reasons: on the one hand, avoiding the hospital environment as a source of contagion risk, and on the other, avoiding the restrictions adopted by several hospitals (i.e., access denied to the partner). The choice of an out-of-hospital birth implied an economic effort for Italian couples. In fact, the national health system provides reimbursement in some regions, but all the costs are not fully covered [35]. Some Italian and Spanish parents had already considered this option before the COVID-19 pandemic, which was not the main, but the decisive, reason for this choice.

Finally, it should be noted that a heterogeneous level of applying recommendations and guidelines emerged. More specifically, it appears that the COVID-19 guidelines for pregnancy and childbirth were promptly applied in hospitals where practices were already compliant with respectful maternity care before the pandemic. In such institutions, excellent solutions to ensure the presence of the partner, skin-to-skin contact, and breastfeeding support were adopted. On the contrary, any lack of support in other hospitals was explained as an organisational issue due to the COVID-19 pandemic. Thus, it was shown how easy it is for reproductive rights and maternal-child healthcare to regress in times of crisis [36]. For this reason, the importance of assuring respectful maternity care both in ordinary conditions and in the pandemic era has been highlighted in many institutions and associations [37,38,39].

### Strengths and Limitations

A better understanding of how the COVID-19 pandemic affected the birth experiences of Italian and Spanish parents was offered in this study. Some differences were found between the Italian and Spanish posts. In general, the concept of obstetric violence emerged more explicitly in the Spanish posts, probably due to socio-cultural factors that make Spanish women more aware of and sensitive about this topic. On the contrary, the denied or limited access of the partner or a trusted person during birth emerged more frequently in the Italian posts. Consequentially, the choice of paying for visits or examinations in private healthcare services that were more organised regarding partner access occurred mostly in Italian posts. The parents who wrote the posts may not represent the whole population due to their limited number. However, it is well established that samples in qualitative research are small [40]. Although the posts were only extracted from Facebook, we believe this has no impact on the representativeness of the results. In fact, the literature shows that Facebook-recruited samples are similarly representative to those recruited traditionally [41]. Moreover, we chose the Facebook pages of the two main associations for respectful maternity care in Italy and Spain. In this regard, it should be noted that most of the shared experiences concerned critical issues and the perception of disrespect for maternal–child rights rather than positive experiences. Furthermore, we were not allowed by the study design and data selection to collect parent socio-demographic data. Additionally, some meanings expressed in the mother tongue could have been lost in the translation.

## 5. Conclusions

Parents who lived with the experience of birth during the COVID-19 pandemic were given a voice by the findings of this study. If, on the one hand, anxiety, fear, and loneliness were the most frequently reported negative feelings, then on the other hand, positive aspects also emerged, including many coping strategies adopted by parents to self-provide a better experience in pregnancy and childbirth. Moreover, especially after the first months of the pandemic, several hospitals maintained high-quality care and met the satisfaction of the parents. From this perspective, hospital preparedness should be oriented to ensure that parents and newborns experience respectful care, even during emergency circumstances.

## Data Availability

Data from this article are available upon request to the corresponding author.
